# Structures of HIV-1 RT-RNA/DNA ternary complexes with dATP and nevirapine reveal conformational flexibility of RNA/DNA: insights into requirements for RNase H cleavage

**DOI:** 10.1093/nar/gku487

**Published:** 2014-05-31

**Authors:** Kalyan Das, Sergio E. Martinez, Rajiv P. Bandwar, Eddy Arnold

**Affiliations:** Center for Advanced Biotechnology and Medicine, Department of Chemistry and Chemical Biology, Rutgers University, Piscataway, NJ 08854, USA

## Abstract

In synthesizing a double-stranded DNA from viral RNA, HIV-1 reverse transcriptase (RT) generates an RNA/DNA intermediate. RT also degrades the RNA strand and synthesizes the second DNA strand. The RNase H active site of RT functions as a nuclease to cleave the RNA strand; however, the structural basis for endonucleolytic cleavage of the RNA strand remains elusive. Here we report crystal structures of RT-RNA/DNA-dATP and RT-RNA/DNA-nevirapine (NVP) ternary complexes at 2.5 and 2.9 Å resolution, respectively. The polymerase region of RT-RNA/DNA-dATP complex resembles DNA/DNA ternary complexes apart from additional interactions of 2′-OH groups of the RNA strand. The conformation and binding of RNA/DNA deviates significantly after the seventh nucleotide versus a DNA/DNA substrate. Binding of NVP slides the RNA/DNA non-uniformly over RT, and the RNA strand moves closer to the RNase H active site. Two additional structures, one containing a gapped RNA and another a bulged RNA, reveal that conformational changes of an RNA/DNA and increased interactions with the RNase H domain, including the interaction of a 2′-OH with N474, help to position the RNA nearer to the active site. The structures and existing biochemical data suggest a nucleic acid conformation-induced mechanism for guiding cleavage of the RNA strand.

## INTRODUCTION

HIV-1 reverse transcriptase (RT) is a central enzyme responsible for copying the viral single-stranded RNA (ssRNA) into a double-stranded DNA (dsDNA) in the cytoplasm of an infected cell (1–3). This event occurs after a virus infects a cell. The synthesized viral dsDNA is transported into the nucleus as a pre-integration complex, and subsequently integrated into the chromosome of the infected cell. Copying of viral RNA to dsDNA involves several steps (4), namely: (i) annealing of a host tRNA^Lys,3^, complementing the primer-binding sequence of the viral ssRNA, forms a double-stranded nucleic acid segment that binds RT to initiate RNA-dependent DNA polymerization; (ii) RT adds nucleotides complementing the (+)RNA strand to synthesize (−)DNA strand starting from the 3′-end of the annealed tRNA; (iii) RNase H activity of RT degrades RNA strand from RNA/DNA hybrid leaving short A-T rich segments, known as polypurine tracts (PPTs), attached to the (−)DNA strand and (iv) the (+)DNA strand synthesis is initiated from the 3′-end of a PPT segment. A non-PPT RNA is usually cleaved in a non-sequence-specific manner. However, the presence of specific nucleotides at positions adjacent to the RNase H site and distal sites in the nucleic acid-binding cleft of RT can enhance RNase H cleavage efficiency (5). The rate of RNase H cleavage is slower than polymerization by RT (6,7). Unlike nucleotide addition by RT, which progresses with incorporation of one nucleotide at a time, the RNase H cleaves discrete phosphodiester bonds of the RNA strand from an RNA/DNA duplex (8–10), i.e. HIV-1 RNase H acts as an endonuclease rather than as an exonuclease enzyme. Biochemical studies have revealed that RT degrades the RNA strand by combinations of primer-dependent primary cuts and primer-independent secondary cuts (11); see reviews by Schultz and Champoux (12) and Beilhartz and Gotte (13). The primary cut of an RNA strand occurs about 18 nucleotides away from the polymerase active site for which the RNA/DNA would occupy the entire nucleic acid cleft extending from the polymerase site to the RNase H site. Sliding of RT over an RNA/DNA substrate (14,15) might facilitate the secondary cleavages. However, the detailed structural bases for both the primary and the secondary cleavages remain elusive.

The DNA polymerization activity of RT has been a central drug target for anti-AIDS therapy. Thirteen RT inhibitors (eight nucleoside/nucleotide inhibitors, NRTIs; five non-nucleoside RT inhibitors, NNRTIs) are approved for treating HIV-1 infection. In contrast, the RNase H activity has not yet been successfully targeted for blocking viral replication. HIV-1 RNase H has a two cation-dependent nuclease activity (16), and the enzyme shares an active site architecture (17) that is conserved in RNase H enzymes in bacteria (18), human (19) and functionally related enzymes like Argonaute (20). The HIV-1 integrase active site also shares a common active site geometry with HIV-1 RNase H. Therefore, both enzymatic activities of HIV-1 are inhibited by common classes of metal-chelating small-molecule inhibitors such as diketo acid derivatives (21–23). Metal-chelating inhibitors have been successfully optimized to develop the integrase-inhibiting anti-AIDS drugs raltegravir, dolutegravir and elvitegravir. However, analogous efforts in optimizing active-site metal-chelating RNase H inhibitors into drug candidates have not yet been successful, presumably due to inability in attaining significant binding specificity and affinity for the compounds against the HIV-1 RNase H site beyond the metal chelation (24–27). Specificity and activity also differ among RNase H enzymes; for example, *Escherichia coli* RNase H is a functionally independent enzyme, whereas the HIV-1 RNase H domain requires additional elements from the polymerase domain for RNase H cleavage activity (28). The RNase H domain of a closely related retrovirus, Moloney murine leukemia virus, was shown to be active; however, the polymerase domain is required for providing specificity for the cleavage of RNA from an RNA/DNA duplex (29,30). Taken together, these studies imply that RNA degradation by HIV-1 RT is a complex process that involves structural elements beyond the RNase H domain to guide the RNA strand to the RNase H active site, even though the mechanism of catalytic cleavage of phosphodiester bonds is conserved among RNase H and related enzymes.

Structurally, RT is a heterodimer of p66 and p51 polypeptide chains. The p66 subunit contains both the polymerase and RNase H active sites. The p51 subunit is proteolytically derived from a p66 chain by HIV-1 protease cleaving off the C-terminal RNase H domain; p51 plays a structural role. RT is a highly flexible enzyme that undergoes conformational changes to carry out its activity. Several conformational and functional states of RT have been structurally characterized (31). While most RT structures are in complexes with an NNRTI or with DNA/DNA that predominantly elucidate DNA-dependent DNA polymerization, inhibition and resistance activities around the polymerase active site, only two publications have reported structures of complexes of HIV-1 RT with RNA/DNA. The structure of a (PPT) RNA/DNA in complex with RT at 3 Å resolution showed base-pairing mismatches in the AT-rich region, and the report suggested that the meltdown may have a role in keeping the RNA strand away from the RNase H active site for this PPT template-primer (32). This study also identified the ‘RNase H primer grip’ as a structural element in p66 that binds the DNA primer strand in the vicinity of the RNase H region. A recent structural study shows a significantly different track for RNA/DNA compared to those in all remaining RT-nucleic acid complex structures (33); the highest resolution structure at 3.3 Å has a nick in the middle of the RNA strand and the other two structures were determined at ∼5 Å resolution. However, in both of the RT-RNA/DNA structures (32,33), the RNA strand does not reach the RNase H active site. Therefore, the structural basis for the mechanism by which an RNA strand reaches the RNase H active site for catalytic RNA degradation remains elusive even 20 years after the first structures of HIV-1 RT were reported (34,35).

**Figure 1.**
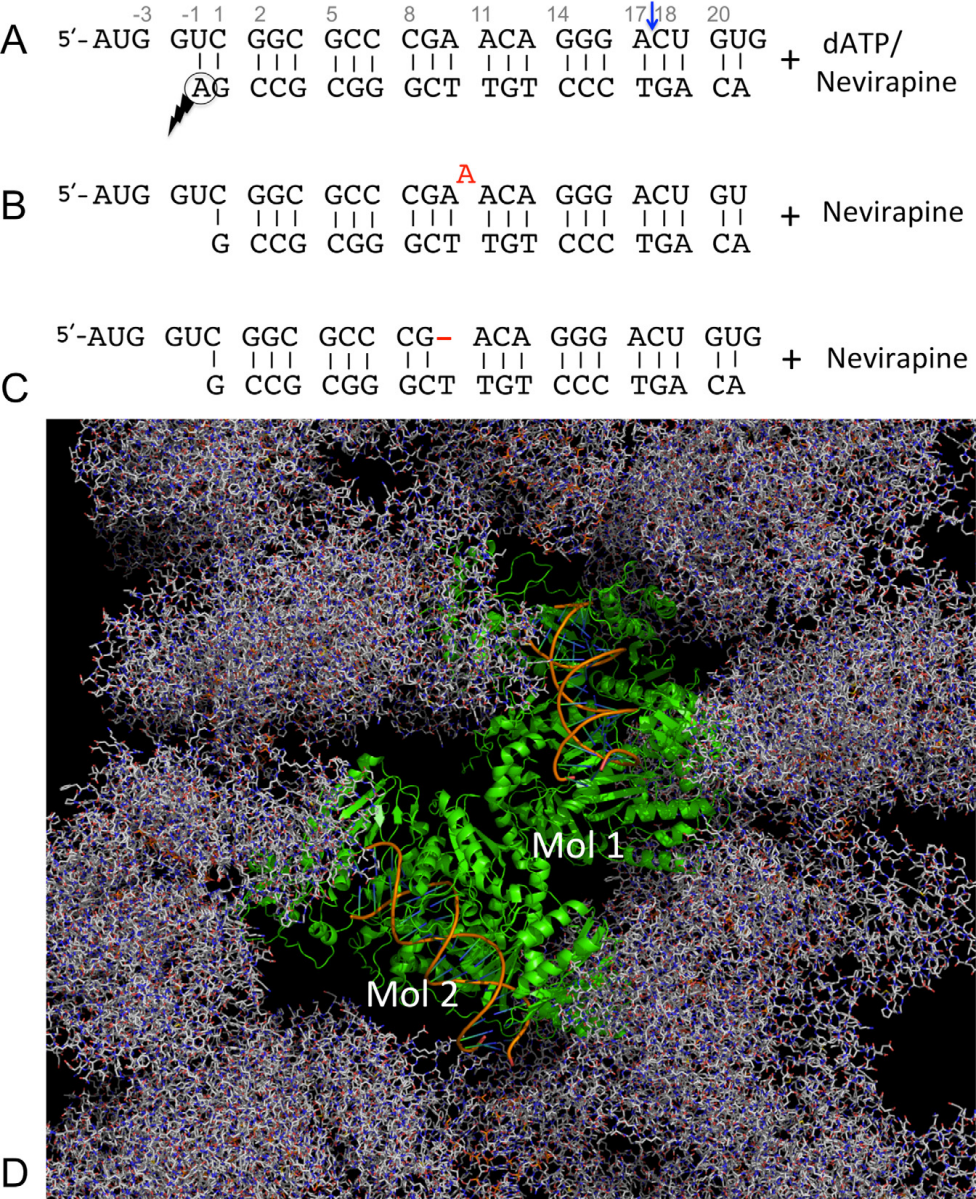
RNA/DNA constructs and the crystal form used in the current study of structures of HIV-1 RT-RNA/DNA complexes. (**A**) Sequences of a 27/21-mer RNA/DNA used in the structural study of RT-RNA/DNA-dATP (or NVP) complex; the nucleotides are numbered at frequent intervals, and the blue arrow indicates the RNase H cleavage position on the RNA strand. A gapped RNA (**B**) and a bulged RNA (**C**) used in the structural studies. The positions of a designed bulge (A) or a gap (−) are shown in red in the two sequences below. (**D**) Crystal packing in the current crystal form shows no significant crystal contacts involving the RNA/DNA template-primers, permitting systematic comparison of conformational changes of RT and RNA/DNA associated with binding of a dNTP or an NNRTI in crystals.

Here we report four structures of HIV-1 RT-RNA/DNA complexes in the presence of either a dATP substrate or an NNRTI drug, nevirapine (NVP); the RNA/DNA sequences used in the current study are shown in Figure 1A–C. The RT-RNA/DNA-dATP structure determined at 2.5 Å resolution, the highest resolution for an RT-nucleic acid complex, defines the binding of an RNA/DNA substrate to RT and reveals distinctions relative to binding of DNA/DNA duplexes. A systematic analysis of this series of RT-RNA/DNA structures reveals multiple conformational states of RNA/DNA, suggesting that a nucleic acid conformation-induced mechanism could be responsible for regulating sporadic cleavages of the RNA strand by RT.

## MATERIALS AND METHODS

### RT expression, RT-RNA/DNA cross-linking and purification of complexes

HIV-1 RT construct RT127A containing an RNase H active site mutation D498N and a thumb mutation Q258C for RT-RNA/DNA cross-linking was cloned, expressed and purified following a previously reported protocol (36). The RNA/DNA template-primer sequences used in the current study are shown in Figure 1. The cross-linkable 20-mer DNA primer was custom synthesized (37), and the RNA templates [27-mer RNA, 27-mer bulged RNA (bulgeRNA) and 12- plus 14-mer gapped RNA (gapRNA)] were synthesized by Dharmacon and Integrated DNA Technologies. Each annealed template-primer was cross-linked to RT127A at the mutated Q258C site of p66, followed by extension with a ddG at the 3′ end through RT polymerization, and the individual complexes were purified using a tandem Ni-NTA and heparin column as previously described (38). To remove the His tag, the RT-RNA/DNA complexes were treated with human rhinovirus 14 3C protease.

### Crystallization and crystal soaking

#### RT-RNA/DNA-dATP crystals

The RT-RNA/DNA binary complex was concentrated to 9 mg/ml in 75 mM NaCl and 10 mM Tris pH 8.0. Crystals were grown at 4°C by hanging-drop vapor diffusion against well solution containing 50 mM bis-tris propane-HCl pH 7.0, 10% PEG 8000 (w/v), 100 mM ammonium sulfate, 5% glycerol (v/v), 5% sucrose (w/v) and 20 mM MgCl_2_. The crystals were taken out of the drop and transferred to a stabilization solution containing 1.5 μl of 50 mM bis-tris propane pH 7.0, 14% PEG 8000 (w/v), 20 mM MgCl_2_, 5% sucrose (w/v), 5% glycerol (v/v) and 100 mM ammonium sulfate for 10 min. The crystals were then treated for 15 min in a solution containing 5 mM dATP, 14% PEG 8000 (w/v) and 1 mM MgCl_2_ for forming the RT-RNA/DNA-dATP complex in crystals. The crystals were cryoprotected by dipping for 1 min into the above solution with glycerol concentration increased to 20% (v/v), and flash frozen in liquid N_2_.

#### RT-RNA/DNA-NVP crystals

The RNA/DNA, gapRNA/DNA and bulgeRNA/DNA complexes were buffer exchanged and concentrated to 12 mg/ml in 75 mM NaCl and 10 mM Tris-HCl pH 8.0. The crystals were grown at 4°C in sitting drops, by vapor diffusion against well solution containing 50 mM bis-tris propane pH 7.2 – 7.4, 9 – 11% PEG 8000 (w/v), 100 mM ammonium sulfate, 5% glycerol (v/v), 5% sucrose (w/v) and 20 mM MgCl_2_.

Crystals of RT-RNA/DNA complex were transferred stepwise into 200 μl of solution I [50 mM bis-tris propane-HCl pH 7.4, 100 mM ammonium sulfate, 5% sucrose (w/v), 10% (v/v) glycerol and 12% (w/v) PEG 8000] for 30 min to remove Mg^2+^ ions, then into 50 μl of solution II [solution I containing 2 mM NVP and 4% DMSO (v/v)] for 3 h 30 min and finally into 10 μl solution III [solution II with glycerol concentration increased to 20%] for 1.5 min. The RT-RNA/DNA-NVP crystals harvested from solution III were flash frozen in liquid N_2_. A similar procedure was followed to obtain the crystals of RT-bulgeRNA/DNA-NVP or RT-gapRNA/DNA-NVP complexes (Figure 1A–C). Two millimolar of CaCl_2_ was added to solutions II and III for the soaking of RT-bulgeRNA/DNA and 2 mM of MnSO_4_ was added to solutions II and III for the soaking of RT-gapRNA/DNA crystals, respectively.

### Data collection, processing and structure determination

Flash-frozen crystals were transported to the F1 beam line at the Cornell High Energy Synchrotron Source (CHESS). X-ray diffraction data were collected from a 280 × 120 × 30 μm^3^ crystal of RT-RNA/DNA-dATP, a 200 × 200 × 60 μm^3^ crystal of RT-DNA/RNA-NVP, a 280 × 120 × 30 μm^3^ crystal of RT-gapRNA/DNA-NVP and a 240 × 120 × 40 μm^3^ crystal of RT-bulgeRNA/DNA-NVP complex. Individual datasets were processed and scaled using HKL2000 (39) (Table 1). The diffraction data were severely anisotropic, and the datasets were corrected for anisotropy using the UCLA MBI – Diffraction Anisotropy Server (40). Atomic coordinates of an RT molecule in RT-DNA/DNA-AZTTP (PDB ID 3V4I) or RT-DNA/DNA-NVP (PDB ID 3V81) complexes (37) were used as starting models to locate the two copies of RT in RT-RNA/DNA-dATP or RT-RNA/DNA-NVP complexes, respectively, by molecular replacement. The initial orientations and positions of individual subdomains (fingers, palm, thumb and connection) and RNase H domain were ascertained by rigid-body refinements of the structural segments of the individual starting models from molecular replacement solutions. The positions and conformations of RNA/DNA, dATP and NVP in the structures were identified and built into difference Fourier maps calculated prior to inclusion of the respective structural elements in refinements. The structures were refined using PHENIX (41) and atomic models were rebuilt using Coot (42) in cycles. Because of noticeable structural differences between the two copies of each complex per crystallographic asymmetric unit (Figure 1D), no non-crystallographic restraints were used in the refinement of any of the structures. Final refinement statistics for the structures are listed in Table 1. The figures were generated using PyMol (http://www.pymol.org/) or ChemBioDraw (http://www.cambridgesoft.com/).

**Table 1.** X-ray data and refinement statistics

	RT-RNA/DNA- dATP	RT-RNA/DNA-NVP	RT-gapped RNA/DNA-NVP	RT-bulged RNA/ DNA-NVP
PDB ID	4PQU	4PUO	4Q0B	4PWD
Data collection				
Space group	P2_1_	P2_1_	P2_1_	P2_1_
Cell dimensions				
*a*, *b*, *c* (Å)	89.44, 128.29, 130.68	89.74, 132.13, 141.96	89.72, 131.05, 141.63	89.68, 130.77, 141.87
α, β, γ (°)	90, 101.76, 90	90, 100.63, 90	90, 100.69, 90	90, 100.75, 90
Resolution (Å)	50.0–2.5 (2.54 –2.5)*	50.0–2.9 (2.95 – 2.9)	50–3.3 (3.36 – 3.3)	50.0 – 3.0 (3.05 – 3.0)
*R*_merge_	0.102 (0.532)	0.093 (0.687)	0.142 (0.670)	0.097 (0.669)
*I* / σ(*I*)	9.7 (1.8)	8.8 (1.3)	7.0 (1.4)	10.3 (1.6)
Completeness (%)	98.4 (96.7)	98.8 (94.3)	98.6 (97.6)	96.2 (90.9)
Redundancy	3.6 (3.0)	3.5 (2.7)	3.0 (2.5)	3.6 (3.0)
				
Refinement				
Resolution (Å)	40.5 – 2.5	46.5 – 2.9	46.4 – 3.3	44.7 – 3.0
Cut-off criteria	*F* ≤ 1.4	*F* ≤ 0	*F* ≤ 0	*F* ≤ 0
No. reflections (*R*_free_ set)	96 925 (2922)	70 967 (2135)	47 845 (1419)	61 963 (1837)
*R*_work_ / *R*_free_	0.202/0.266	0.228/0.285	0.237/0.317	0.221/0.291
No. atoms:				
Protein+DNA	17 300	17 427	17 3781	17 461
Ligand/ion/solvent	60/4/450	40	40/2/5	40/2	
Stereochemistry (RMSDs)				
Bond lengths (Å)	0.01	0.015	0.013	0.015
Bond angles (°)	1.26	1.79	1.77	1.65

One crystal was used for each data set. *Values in parentheses are for highest-resolution shell.

## RESULTS

### Structure of RT-RNA/DNA-dATP complex

**Figure 2.**
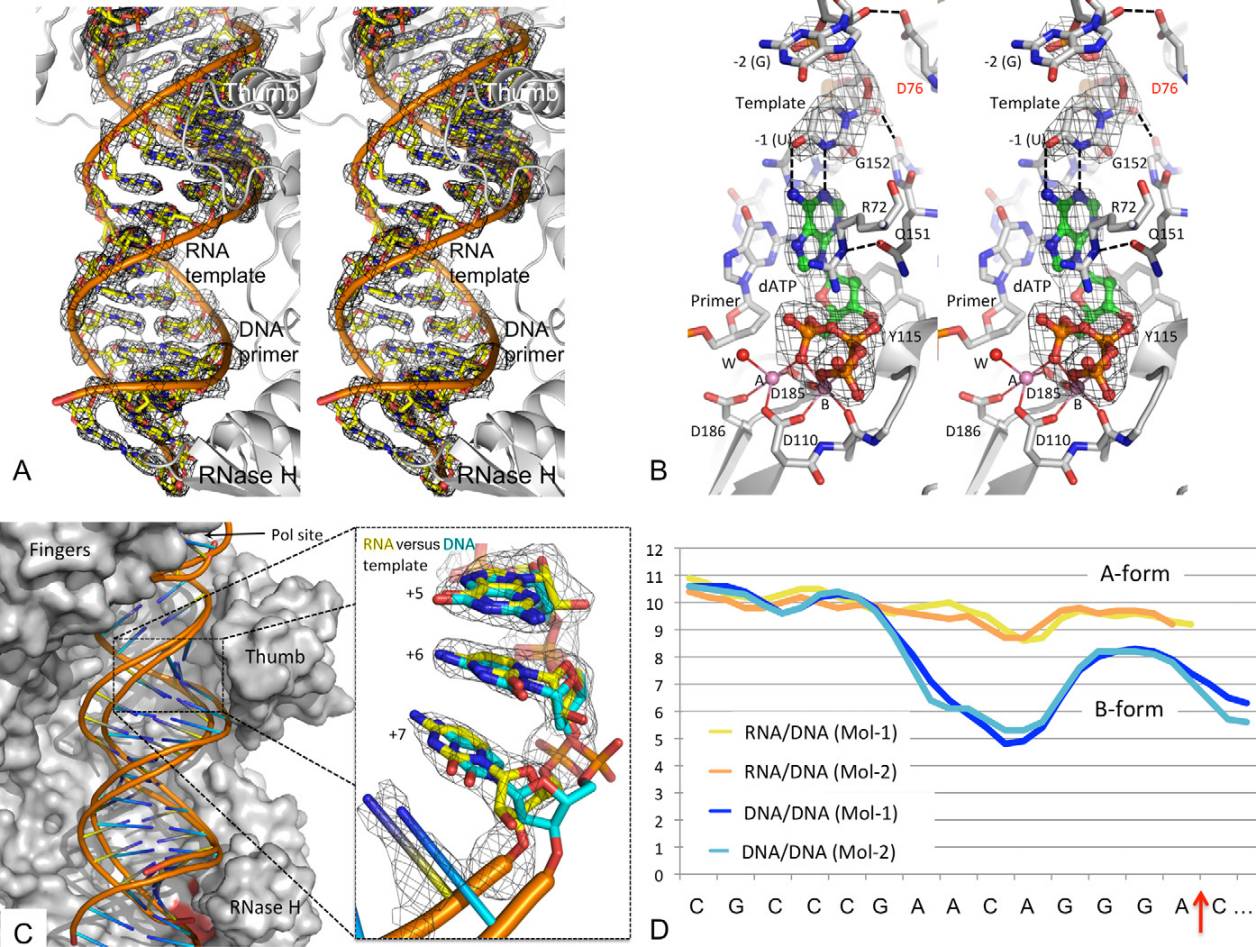
Structure and conformation of RT-RNA/DNA-dATP complex. (**A**) A stereoview of the electron density (2Fo-Fc) calculated at 2.5 Å resolution defines the binding track and conformation of RNA/DNA bound to RT. (**B**) A stereoview showing the binding of dATP in a polymerase catalytic mode. The binding involves chelation of two Mg^2+^ ions (A and B) with the catalytic aspartic acid residues (D110, D185 and D186); the metal ions chelate all three phosphates of dATP (green). ‘W’ represents the only water molecule involved in the metal chelation. Use of a 3′-dideoxy-terminated primer prevents the catalytic incorporation of the nucleotide. (**C**) Comparison of the modes of binding of RNA/DNA (yellow spokes) versus DNA/DNA [cyan spokes, PDB ID. 3V4I (37)] to HIV-1 RT (gray surface) in the respective catalytic ternary complexes shows significant deviations in nucleic acid binding tracks away from the polymerase active site. The structures of RT-RNA/DNA-dATP and RT-DNA/DNA-dATP [PDB ID. 3V4I (37)] were aligned by superimposing Cα atoms of RT. A zoomed view of the RNA template illustrates the differences in sugar conformations and phosphate positions after the fifth nucleotide from the polymerase active site. Differences in sugar ring conformations between RNA versus DNA template account for the divergent binding tracks of RNA/DNA (yellow) versus DNA/DNA (cyan) following the seventh nucleotide. (**D**) Minor groove width (y-axis in A) analysis of the two independent copies of DNA/DNA (blue and cyan) versus two independent copies of RNA/DNA (yellow and orange) *in crystallo* using CURVES+ (53) shows that the RT-bound RNA/DNA maintains A-like conformation, whereas the DNA/DNA deviates away from A-like conformation beyond the polymerase site. The RNase H cleavage site is indicated by a red arrow on the x-axis.

RT undergoes conformational changes involving hinge motions between subdomains for carrying out its multiple functions. Crystal structures have revealed the structural and conformational changes of RT upon binding of a double-stranded nucleic acid, a dNTP or an NNRTI (31). We have discovered a crystal form of RT-nucleic acid complex (Figure 1D) in which RT molecules are well separated by disordered solvent regions (channels), permitting visualization of large conformational changes and hinge motions of RT in crystals (37). This flexibility of RT molecules in a crystal permitted (i) the binding of a dNTP to form a catalytic RT-RNA/DNA-dATP polymerase complex or (ii) the binding of NVP to form NNRTI-inhibited RT-RNA/DNA-NVP ternary complex. The structures of the dATP- and NVP-ternary complexes were determined at 2.5 and 2.9 Å, respectively. 2.5 Å resolution corresponds to the highest detail for an RT-nucleic acid complex structure reported so far, permitting reliable definition of the position, conformational state and interactions of individual nucleotides of the RNA/DNA substrate (Figure 2A).

In the RT-RNA/DNA-dATP complex, the dATP substrate binds near the polymerase active site in a mode similar to that in an analogous RT-DNA/DNA-dATP complex, earlier obtained in a different crystal form (43). Additionally, the current structure reveals the binding of dATP involving two divalent cations that chelate three catalytic aspartates (D110, D185 and D186); the side chain of the third catalytic aspartate (D186) is involved in chelation of metal ion A (Figure 2B). An RNA/DNA binds RT with a higher affinity than a DNA/DNA template-primer, attributable to the additional interactions of 2′-OH groups of the RNA template with the polymerase domain (44). In the crystal structure, (i) the 2′-OH of the first nucleotide overhang (-1; Figure 1A), complementing the dATP, forms a hydrogen bond with the main-chain carbonyl oxygen of G152; (ii) residues Q151 and G152 form a part of the dNTP-binding pocket (Figure 2B) and (iii) the 2′-OH of the second nucleotide overhang (–2) interacts with the side chain of D76, a conserved residue at the base of the functionally critical β3−β4 sheet that rearranges to assist dNTP binding. These additional interactions of RNA with the ‘template grip’ (35) may also account for the higher fidelity of RNA-dependent DNA polymerization compared to DNA-dependent DNA polymerization by RT (45,46). These interactions between the 2′-OH groups of RNA template and RT are also conserved in a recently reported structure of an RNA/DNA in complex with the polymerase domain of Moloney murine leukemia virus (47).

Apart from the 2′-OH interactions, the nucleic acid and dNTP superimpose well at and around the polymerase active site when the RNA/DNA complex is compared with RT-DNA/DNA-dNTP ternary complexes (37,43,48–49). However, moving towards the RNase H active site, the nucleic acid track for RNA/DNA deviates significantly from DNA/DNA (Figure 2C): the minor groove width suggests an A-like form for RNA/DNA and a B-like form for DNA/DNA in this region. The sugar-phosphate backbone of the first five nucleotides from the polymerase active site of both the RNA and DNA templates superimposes, and the sugar rings in both have C3′-endo (A-form) conformation. The puckering of the sugar rings and consequently the positions of phosphates start diverging from the seventh nucleotide (Figure 2C); the ribose rings of the RNA template in the RT-RNA/DNA-dATP complex maintain C3′-endo conformation, whereas the deoxyribose rings of the DNA template start switching conformation away from C3′-endo. The average inter-base-pair parameters (Supplementary Table S1), however, suggest no distinct differences between the RT-bound RNA/DNA and DNA/DNA conformations.

The two copies of the RT-RNA/DNA-dATP complex in the crystal asymmetric unit (Figure 1D) have similar overall structure and RNA/DNA conformation; however, the local structures and thermal vibrations (B-factors) differ, reflecting significant flexibility and adaptability of RT and RNA/DNA captured by the current crystallography experiments (Supplementary Figure S1). The RNA/DNA duplex maintains A-form geometry, whereas the conformation of DNA/DNA duplex in an analogous crystal form (37) differs for the stretch extending towards RNase H active site (Figure 2D). In contrast to an A-like conformation of regular RNA/DNA in the current structure, the (PPT) RNA/DNA in the earlier published structure (32) has non-A form geometry in this region, which may be a reason that RT does not recognize a (PPT) RNA/DNA as a substrate for RNase H cleavage. However, the scissile phosphodiester bond is not positioned appropriately at the RNase H active site of the current 2.5 Å RT-RNA/DNA-dATP structure, and thus does not represent the conformational state of an RNase H catalytic complex. The structure reveals a polymerase-competent mode of RT using an RNA/DNA template-primer; however, the RNA/DNA may need to undergo conformational changes to interact with the RNase H active site for cleavage of the RNA strand. Binding of an NNRTI enhances RNase H cleavage activity (14), therefore it is plausible that the RNA strand may be guided towards the RNase H active site in the structure of an RT-RNA/DNA-NNRTI complex.

### Structure of RT-RNA/DNA-NVP complex

Binding of NVP to RT-RNA/DNA complex was accompanied by significant changes, predominantly involving rearrangements of the subdomains and repositioning of the RNA/DNA away from the polymerase active site. Analogous to what was observed in the formation of an RT-DNA/DNA-NVP complex (37), the RNA/DNA duplex is shifted away from the polymerase active site; consequently, the distorted dNTP-binding site does not accommodate a dNTP. Consistent with these observations, a recent isothermal titration calorimetry study demonstrated the inability of an RT-DNA/DNA-NNRTI complex to bind a dNTP substrate in solution (50).

**Figure 3.**
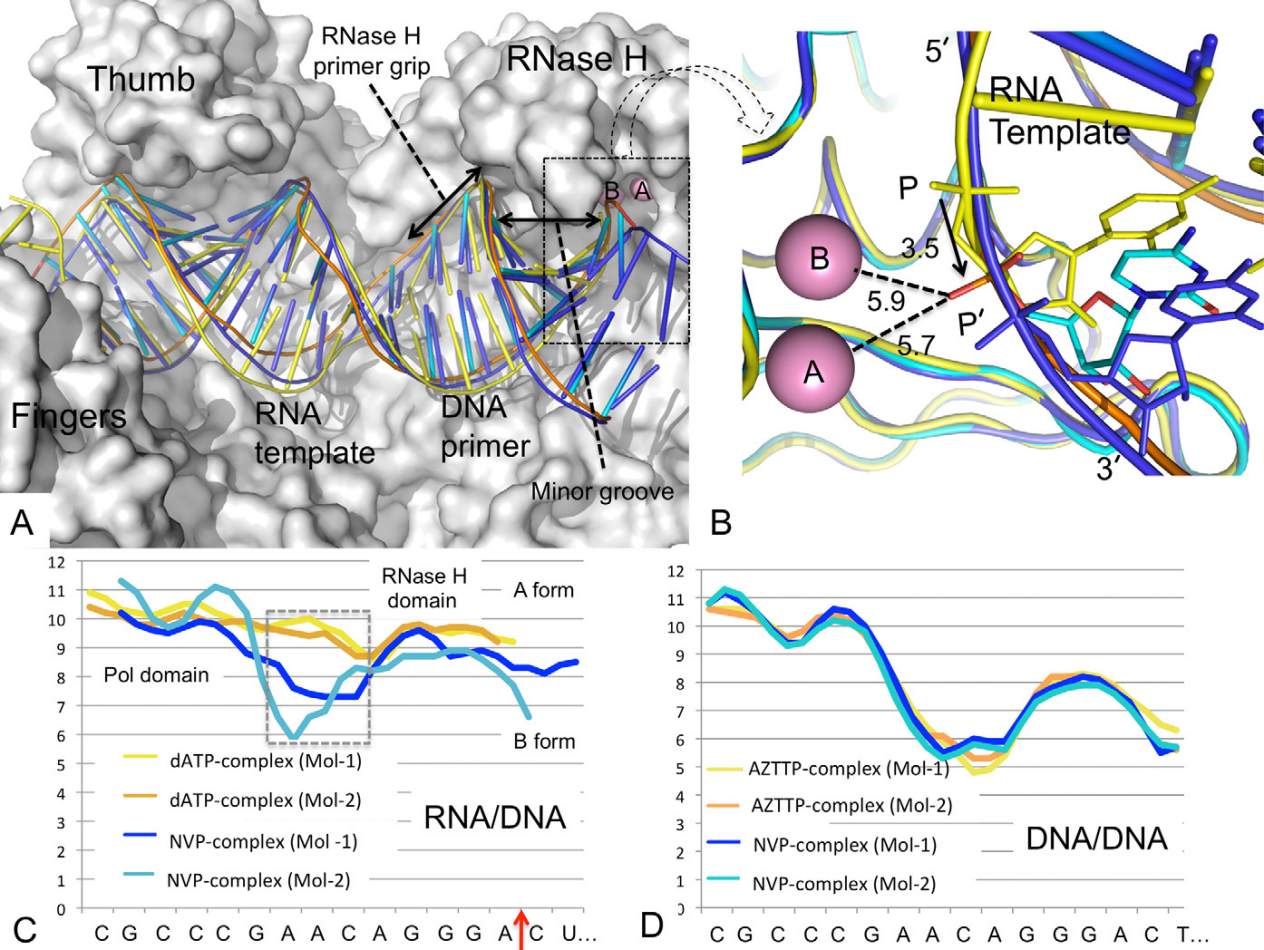
Conformational changes of RNA/DNA upon binding of NVP to RT. (**A**) Comparison of the binding tracks of RNA/DNA in RT-RNA/DNA-dATP complex (Mol-1; yellow) and the two independent copies in RT-RNA/DNA-NVP (Mol-1, blue; Mol-2, cyan); the molecular surface of RT in Mol-2 is shown in gray. (**B**) A closer view of the RNase H active site of panel A shows the shift of scissile phosphate towards the RNase H active site, represented by two metal ions A and B. The metal ions are positioned by aligning the RNase H domain of the structure RT-β- thujaplicinol (PDB ID 3IG1) (24) binary complex with the current structures. The scissile phosphate in the RT-RNA/DNA-NVP complex (Mol-2) is shifted by ∼3.5 Å from P to P’, and P’ is positioned almost equidistant from the metal ions A and B; the distances are listed in A. (**C**) The CURVES+ () analysis of minor groove width (y axis in A) of RNA/DNA in the two independent copies of RT-RNA/DNA-dATP (yellow and orange) and RT-RNA/DNA-NVP (blue and cyan) structures; the RNA/DNA conformation superimposes well in RT-RNA/DNA-dATP copies, however, deviates upon NVP binding. (**D**) In contrast to RNA/DNA, the conformation of DNA/DNA is influenced significantly less by NNRTI binding (53). The minor groove widths of two independent copies of DNA/DNA in RT-DNA/DNA-NVP (yellow and orange; PDB ID 3V81) and RT-DNA/DNA-AZTTP (blue and cyan; PDB ID 3V4I) structures by CURVES+ are plotted.

When the two copies of RT-RNA/DNA-NVP complex in the crystal are compared, the NVP binding site is highly superimposible; however, the RT subdomains are rearranged a little differently in one copy (Mol-1) compared to the other (Mol-2). Differences in subdomain arrangements are also observed from comparison of multiple crystal structures of RT/NVP binary complex; e.g. PDB IDs 3HVT (34) versus 1RTH (51). In the current RT-RNA/DNA-NVP structure, the differences in the arrangements of the subdomains impacted the conformation of RNA/DNA in the two molecules differently. As a consequence, the RNA strands have conformational differences and are positioned differently with respect to the RNase H active site in Mol-2 compared to that in Mol-1 of RT-RNA/DNA-NVP complex in the crystal (Figure 3A–C). In contrast, the conformation of DNA/DNA (Figure 3D) is less perturbed upon binding of NVP when previously reported structures of RT-DNA/DNA-NVP (PDB ID 3V81) and RT-DNA/DNA-AZTTP (PDB ID 3V4I) obtained in an analogous crystal form were compared (37).

In structures of HIV-1 RT in complexes with RNase H inhibitors, the inhibitors chelate both RNase H active site cations (24–26). We aligned the RNase H domain of the HIV-1 RT/β- thujaplicinol structure (PDB ID 3IG1) (24) to position the two cations approximately at the RNase H active site of the current RT-RNA/DNA-NVP structure. Based on the above superposition of RNase H domains, the 18th template phosphate (Figure 1A) is positioned almost equidistantly from the two cations (Figure 3B); a phosphate oxygen is positioned ∼5.7 and 5.9 Å away from both metal ions. In light of these considerations, bending of the RNA/DNA towards the active site would be required to bring the phosphate oxygen closer (∼2.5 Å) for chelation with the metal ions. This could be achieved by conformational changes of RNA/DNA, as discussed later.

Protein–nucleic acid interaction and crystallographic B-factor analyses of dATP and NVP complexes suggest that the binding of RNA/DNA to RT is primarily defined by its interactions with palm, thumb and finger subdomains in the polymerase domain, and with the RNase H primer grip in and nearby the RNase H domain (32). The parts of RNA/DNA that interact with RT have relatively lower B-factors (Supplementary Figure S1), and non-interacting parts have higher flexibility and higher B-factors despite the fact that RT-RNA/DNA homogenous complex was formed by cross-linking the DNA primer with RT, i.e. RT and RNA/DNA retain conformational flexibility in the RT-RNA/DNA cross-linked complex used in the current study.

The RNase H primer grip is important for degradation of RNA by RT, and mutations of the primer grip residues cause significant loss of RNase H cleavage activity (52). When compared with the dATP ternary complex, the phosphate group slides laterally ∼3.5 Å towards the RNase H active site upon binding of NVP (Figure 3B) in the NVP ternary complex. The sliding is not uniform, however, for both the RNA template and DNA primer strands (Figure 3A and B). Interactions with the RNase H primer grip stabilize the DNA strand; the interacting part of the DNA has the lowest B-factors compared to the remaining parts of the RNA/DNA in the NVP complex (Supplementary Figure S1). A non-uniform slide is apparently responsible for the base-pair meltdown and conformational distortion of RNA/DNA positioned in the region between the polymerase and RNase H sites where RNA/DNA does not have significant interaction with RT. Analysis of nucleic acid duplex conformations by CURVES+ (53) reveals significant deviation of RNA/DNA conformation from A-form (observed in the dATP-complex) for the region in the NVP complex (Figure 3C). In contrast, NVP binding has significantly less impact on DNA/DNA conformation in the structures obtained earlier in an analogous crystal form (37) (Figure 3D). This structural finding suggests that an RT-bound RNA/DNA can undergo conformational changes induced by binding of an NNRTI or by frequent sliding of RT over RNA/DNA in the process of nucleotide incorporation and translocation. In a biochemical study of an RT-RNA/DNA-foscarnet ternary complex, in which the RNA/DNA slides forward to N site (nucleotide-binding site), the RNase H active site accesses the 19th phosphate rather than the 18th phosphate of an RT-RNA/DNA complex (54).

An RNA strand of an RNA/DNA duplex is cleaved periodically. The template RNA strand does not consistently interact with RT near the RNase H domain, and thereby no structural element of RT continuously threads the RNA to the RNase H active site for the cleavage of phosphodiester bonds. It seems likely that an RNA/DNA duplex constantly undergoes conformational changes while RT slides over it (14,15), and conformational changes of an RNA/DNA may help the RNA strand to reach the RNase H active site. To investigate the structural requirements for an RNA strand of RNA/DNA to be cleaved, and to visualize conformational changes of RNA/DNA that can occur, we introduced a bulge and a gap in the RNA strand as described below.

### Structures of RT bulged (or gapped) RNA/DNA-NNRTI complexes

To artificially introduce conformational variations and investigate the effects on the proximity of the RNA strand with respect to the RNase H active site, we introduced (i) a single-nucleotide bulge and (ii) a single-nucleotide gap in the RNA strand of the RNA/DNA duplex (Figure 1B and C). Following the experimental procedures used for obtaining the previously described complexes, crystals of RT-bulged RNA/DNA (bulgeRNA) and RT-gapped RNA/DNA (gapRNA) complexes were obtained in the described crystal form (Figure 1D), permitting the formation of respective NVP complexes by soaking the NNRTI into crystals as outlined in the Materials and Methods section. Like in the RT-RNA/DNA-NVP complex, the bulged RNA/DNA (or the gapped RNA/DNA) template-primers have conformational differences when the two copies (Mol-1 and Mol-2) in the crystallographic asymmetric unit of a bulgeRNA (or gapRNA) complex are compared (Supplementary Figure S2). In crystals, the RNA/DNA conformation and binding exhibit wider variations in Mol-2 than in Mol-1 even though both copies undergo large conformational changes upon binding of an NNRTI. Hereafter, we will primarily compare and discuss Mol-2 structures of the RNA/DNA complexes unless otherwise stated.

**Figure 4.**
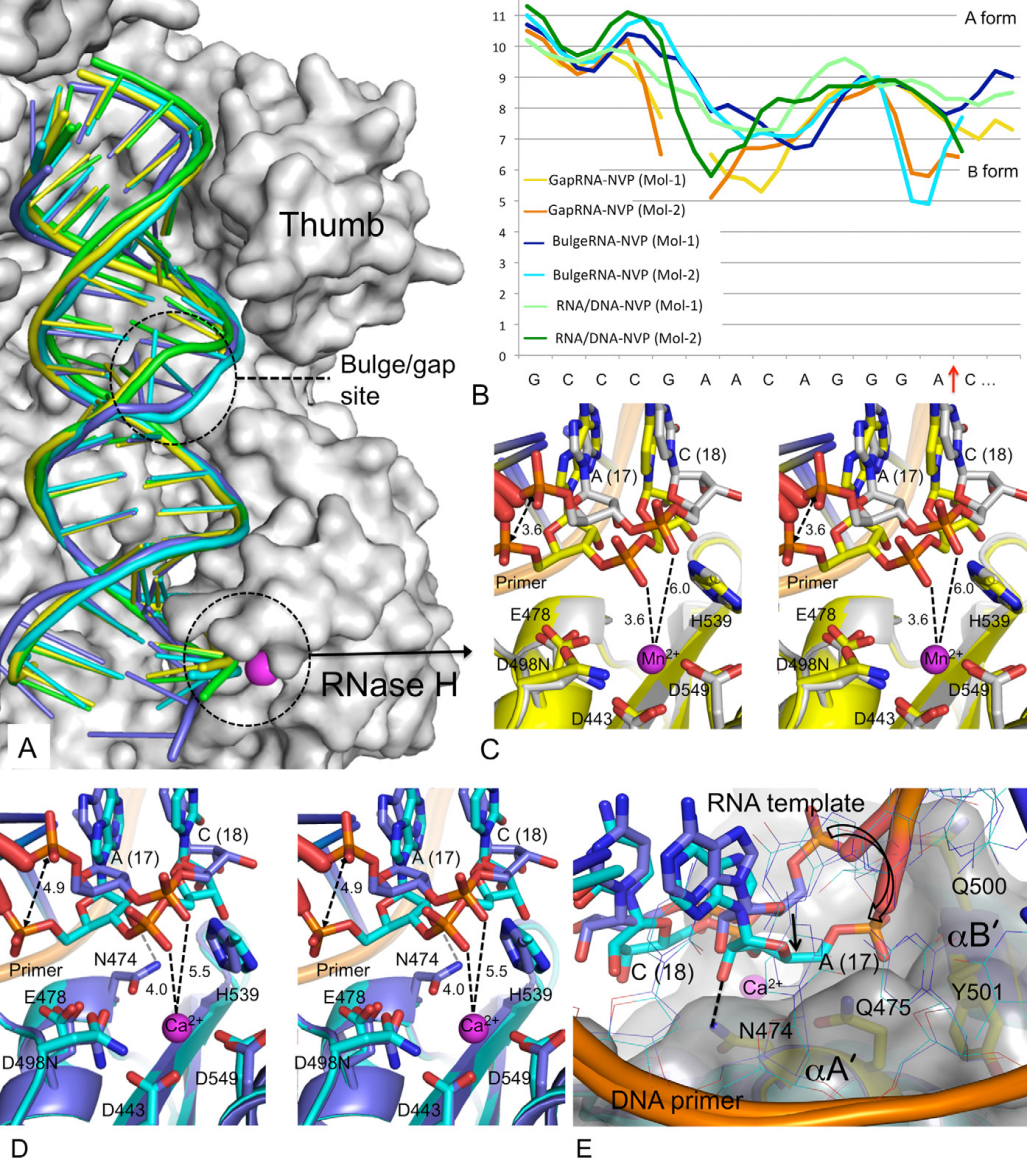
Binding of gapRNA/DNA and bulgeRNA/DNA to HIV-1 RT in respective RT-RNA/DNA-NVP complexes. (**A**) Binding modes of RNA/DNA (green), gapRNA/DNA (yellow) and bulgeRNA/DNA (Mol-1, blue; Mol-2, cyan). The structures were aligned based on RNase H superposition (residues 440 – 540). (**B**) CURVES+ (53) analysis of minor groove width (y axis in A) of two copies of RNA/DNA in structures of the three RT-RNA/DNA-NVP complexes. (**C**) A stereoview showing comparison of binding modes of gapRNA/DNA in Mol-1 (gray) versus Mol-2 (yellow). The RNA strand switches conformation in Mol-2 to permit closer access the RNase H active site. A cation (Mn^2+^) was found bound to the active site. The side chains of conserved RNase H active site residues are shown, and important distances are indicated in Å. (**D**) A stereoview showing comparison of binding modes of bulgeRNA/DNA in Mol-1 (blue) versus Mol-2 (cyan) show conformational changes of RNA in Mol-2 leading to closer approach to the RNase H active site, analogous to gapRNA/DNA in panel C. (**E**) A view of the above superposition of bulgeRNA/DNA (Mol-1 and Mol-2) from the primer grip shows enhanced interactions of the RNA strand of Mol-2 with RNase H helices (αA’ and αB’) including a hydrogen bond of a 2′-OH group with N474.

The structures of gapRNA-NVP and bulgeRNA-NVP complexes were determined at 3.2 and 3.0 Å resolution, respectively (Table 1). As designed, the gap and the bulge on the RNA strands are in the region that does not interact with RT (Figures 1B, C and 4A). The gapRNA or bulgeRNA structures show variations in the RNA/DNA conformation when compared with each other or with the RT-RNA/DNA-NVP complex (Figure 4B). The introduced conformational perturbations impact the positioning of the RNA strand adjacent to the RNase H active site. Interestingly, both a gap and a bulge in the RNA strand help move the scissile phosphate group closer towards the RNase H active site (Figure 4C and D).

**Figure 5.**
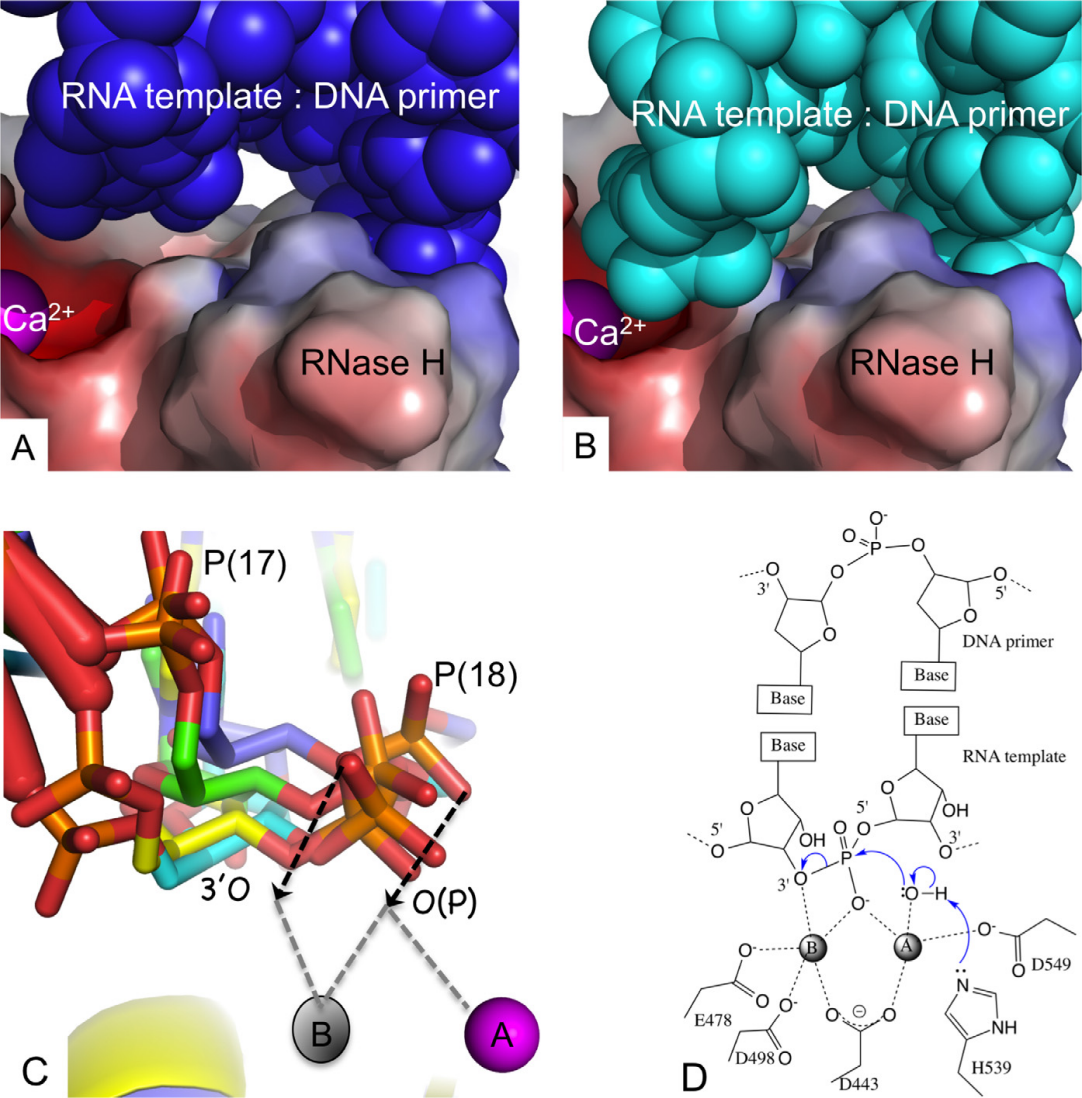
Structural requirements for an RNA strand to be cleaved by RNase H of HIV-1 RT. (**A**) The position of bulgeRNA/DNA (blue CPK model) with respect to the chelated cation (Ca^2+^ ion at metal A position, magenta) at the RNase H active site of Mol-1; the RNase H domain of RT is represented with an electrostatic potential surface. (**B**) The RNA/DNA (cyan CPK model) is folded towards the RNase H active site in Mol-2. (**C**) Superposition of gapRNA (Mol-1, green; Mol-2, yellow) and bulgeRNA (Mol-1, blue, Mol-2, cyan) at the RNase H active site defines a putative trajectory (shown by black arrows) for the RNA of RNA/DNA duplex reaching the RNase H active site for cleavage. The location of cation B is positioned based on a superposition of the RNase H domain of RT-β- thujaplicinol structure (PDB ID 3IG1)(24). (**D**) A general catalytic mechanism of cleavage of a phosphodiester bond by the RNase H activity of HIV-1 RT derived from structural information about nuclease cleavage (16), RNase H cleavage (18) and metal chelation at the HIV-1 RNase H active site (24). Two additional water molecules and one water molecule would chelate metal ions A and B, respectively, to complete octahedral coordination environments for both cations.

Mg^2+^ ions are the cofactors for RNase H activity *in vivo*. However, structures of inhibitors chelated at the RNase H active site of RT could only be obtained in the presence of Mn^2+^ ions (24–26), despite many attempts to obtain structures with Mg^2+^. Mn^2+^ or Ca^2+^ have been substituted for Mg^2+^ in structural studies of RNA/DNA-bound complexes of related enzymes (19). In our attempts to generate and visualize metal chelation at the RNase H active site, we introduced Mn^2+^ or Ca^2+^ ions into the complexes. Mn^2+^ or Ca^2+^ ions were introduced into the crystals of gapRNA or bulgeRNA complexes, respectively, by soaking, as described in Materials and Methods. Only one ion (Mn^2+^ or Ca^2+^) is observed bound at position A at the RNase H active site, and no binding was observed at position B (24). The scissile phosphate is positioned at an interacting distance of ∼3.6 Å from an Mn^2+^ ion and ∼4.0 Å from a Ca^2+^ ion bound at the RNase H active site in the gapRNA and bulgeRNA complexes, respectively. This closer proximity of the RNA strand is accompanied by a significant shift of the 17th template phosphate (Figure 4C and D) and narrowing of the RNA/DNA minor groove adjacent to the RNase H active site (Figure 4B). The phosphates of the DNA primer strand interacting with the RNase H primer grip, however, are relatively unperturbed (Figure 4A). A closer approach of the RNA strand over the RNase H active site is accompanied by enhanced interactions (i) of the 2′-OH of the 17th nucleotide with the side chain of the RNase H primer grip residue N474 and (ii) of the phosphate group of the 17th nucleotide with Y501 of helix αB’ (17) (Figure 4E). Residue N474 is a part of the RNase H helix αA’ that contains the catalytic residue E478, and residues T473 and Q475 (both interact with the DNA primer). The mutation N474A reduces RNase H cleavage, and the double mutation N474A+Q475A has an additive effect (52). Both RNase H helices αA’ and αB’ are positioned at the minor groove of RNA/DNA adjacent to the RNase H active site. The minor groove width is significantly reduced when the RNA strand approaches the RNase H active site, as observed in Mol-2 of gapRNA and bulgeRNA structures (Figures 4B and 5A, B). These structural findings suggest that certain conformational states of RT-RNA/DNA complex enhance the interaction between the RNA strand and the RNase H helices such that the RNA strand approaches the RNase H active site more closely.

### Conformational changes and enhanced RNase H interactions of RNA/DNA facilitate cleavage

The extensive interactions of both the template and primer strands of a nucleic acid with the polymerase domain precisely position the primer 3′-end at the polymerase active site for catalytic incorporation of each nucleotide. In contrast, we did not find any structural element of RT that would systematically thread the RNA strand to the RNase H active site. Preferred nucleotides at positions adjacent and distal to RNase H site enhance RNA cleavage (5), presumably by influencing the conformation of RNA/DNA to access the active site.

Understanding of how the RNA strand of an RNA/DNA duplex is guided to the RNase H active site would illuminate the structural basis for RNA cleavage. The current structures reveal that the dangled RNA strand over the RNase H domain is primarily supported by base pairing and base stacking with the complementing DNA primer, which is held by the conserved interactions with the RNase H primer grip (32). Thereby, the sugar-phosphate backbone of the RNA strand can be rearranged by altering the conformation of the RNA/DNA duplex. Comparison of Mol-1 and Mol-2 in bulgeRNA-NVP (or gapRNA-NVP) crystals reveals such rearrangements of the RNA strand near the RNase H active site (Figure 5A and B). The closer approach of the RNA strand in Mol-2 is accompanied by reduced minor groove width (Figure 4B) and enhanced interactions with the two RNase H helices αA’ and αB’ (Figure 4E).

Structural studies of prototype foamy virus integrase have revealed almost identical positions and chelation environments for two active site cations when the structures of intasome and drug complexes were compared (23). HIV-1 RNase H has an analogous active site architecture and inhibitor-chelation environment. Superposition of RNase H domains of an RT-RNase H inhibitor complex (24) with the current structures provides a logical positional and chelation environment of two divalent cations at the RNase H active site (Supplementary Figure S3). The RNase H superpositions (residues 440–540) of the RT-RNA/DNA structures, together with the positioning of two catalytic metal ions, show multiple positions for the scissile phosphodiester bond and the phosphate group of the 18th nucleotide, apparently mimicking a trajectory for approach to the RNase H catalytic site (Figure 5C); the chelating phosphate oxygen and the preceding 3′-*O* are concurrently approaching the RNase H active site. Presumably, the negatively charged phosphate moiety nearing the RNase H active site would favor chelation of two cations, and cleavage would occur if the RNA strand is oriented and positioned appropriately for catalysis (Figure 5D). In fact, binding of the RNase H inhibitor β- thujaplicinol, which has no significant interactions beyond the active site, is predominantly guided and stabilized by its interaction with both active site metal ions (24). The chelating groups of the RNA strand would be attracted via the metal ions to the RNase H active site and bind in an analogous fashion.

Catalytic cleavage of the phosphodiester bond alters the chelation environment and charge distribution at the RNase H active site, and consequently, the cleaved RNA products disengage from the RNase H active site even if the RNA segments at either end of the cleaved phosphodiester bond remain hybridized to the DNA template. The presence of the D498N mutation or a non-chelating rotameric conformation of E478 (Supplementary Figure S3) might be preventing the formation of a catalytic complex in the current structures. Another structural constraint in nearly all crystal structures of RT is the formation of an extended β-sheet involving two RNase H domains of adjacent molecules. This extended sheet may also restrict the flexibility of RT to attain an RNase H catalytic complex in crystals.

## DISCUSSION

RT is highly dynamic in solution. As revealed by a set of single-molecule fluorescence resonance energy transfer studies, conformational heterogeneity of an RT-nucleic acid complex is (i) increased by the binding of an NNRTI and (ii) decreased by the binding of a dNTP (15). All structures of RT-RNA/DNA complexes reported in the current study are obtained in highly similar chemical and crystallographic environments, and the RT molecules in the crystals are well separated by disordered solvent regions, permitting significant conformational changes required for binding of (i) a dNTP to form a catalytic polymerase complex or (ii) an NNRTI to form a polymerase-inhibited complex. Thereby, the observed structural and conformational changes of an RT-RNA/DNA complex are influenced minimally by the use of analogous experimental environments, and thus can be attributed to dNTP or NNRTI binding. Interestingly, two independent copies of RT-RNA/DNA complexes (Mol-1 and Mol-2) in crystals (Figure 1D) exhibit (i) a relatively homogenous conformational state in the dATP-bound structure and (ii) noticeable differences in NVP-bound structures (Figure 3C). Binding of an NNRTI causes significant domain rearrangements, which enhance sliding of the RNA/DNA substrate such that the scissile phosphodiester bond has better access to the RNase H active site (Figure 3B). The sliding of RNA/DNA over RT is not uniform for both strands, contributing to conformational variations of the RNA/DNA (Figure 3A–C). The designed bulge and gap in RNA introduce additional conformational perturbations to an RNA/DNA duplex that might mimic some of the conformational states accompanying uneven sliding motions of RNA/DNA over RT. Despite large variations in the conformation and binding of RNA/DNA to RT in the presence of NVP, the interactions of the DNA primer strand with the RNase H primer grip remain conserved among the structures. However, the positions of the complementing nucleotides of the RNA strand vary when the structures of RT-RNA/DNA-NVP complexes are compared (Figure 4). Conformational changes of RNA/DNA bring a phosphate oxygen closer to one Ca^2+^ (or Mn^2+^) ion present at the RNase H active site in bulgeRNA or gapRNA structures (with a minimum distance of ∼4 Å between the oxygen and ion) (Figure 4C and D). Closer approach of the RNA strand towards the RNase H active site is accompanied by enhanced interaction of the minor groove of RNA/DNA with the RNase H domain (Figure 5A and B). Comparison of relative positions of the RNA strands in the structures of RNA/DNA complexes reported here shows multiple positions of the scissile phosphate with respect to the RNase H active site, and these positions reveal a possible path for the RNA strand to reach the active site for cleavage (Figure 5C and D).

A recently reported nicked RNA/DNA-RT-efavirenz structure shows a significant deviation in the binding of the RNA/DNA compared to the current structures (Supplementary Figure S4A) and all RT-nucleic acid complexes previously described in the literature; the authors proposed that enhanced interaction between the p51 C-terminus and RNA strand might be critical for RNase H degradation (33). Our analysis of the structure (PDB ID 4B3O)(33) suggested that the nicked RNA/DNA is significantly impacted by extensive crystal contacts (Supplementary Figure S4B), and the interactions of a symmetry-related RT molecule with RNA/DNA apparently have a major influence on defining the altered track of the RNA/DNA duplex approaching the RNase H domain (Supplementary Figure S4A).

The current series of structures provide insights into how the non-uniform sliding of RNA/DNA, and conformational changes of the duplex near to and distal from the RNase H active site assist the RNA strand in accessing the active site for cleavage (5,52,54). Further biochemical and structural experiments guided by the current structures would help further exploration of binding specificity of RNA and inhibitors to the RNase H active sites, and may aid design of new RNase H inhibitors.

## ACCESSION NUMBERS

The atomic coordinates and structure factors for the structures of RT-RNA/DNA-dATP, RT-RNA/DNA-NVP, RT-gapRNA/DNA-NVP and RT-bulgeRNA/DNA-NVP complexes are deposited in Protein Data Bank (PDB) under accession numbers 4PQU, 4PUO, 4Q0B and 4PWD, respectively.

## SUPPLEMENTARY DATA

Supplementary Data are available at NAR Online.

SUPPLEMENTARY DATA
