# Supplementary material for: Structures of HIV-1 RT-RNA/DNA ternary complexes with dATP and nevirapine reveal conformational flexibility of RNA/DNA: insights into requirements for RNase H cleavage
Source: Nucleic Acids Res. 2014 May 31;42(12):8125–37. doi: 10.1093/nar/gku487 (PMC4081091; doi:10.1093/nar/gku487)
Supplement: SUPPLEMENTARY DATA [file supp_42_12_8125__index.html]

Structures of HIV-1 RT-RNA/DNA ternary complexes with dATP and nevirapine reveal conformational flexibility of RNA/DNA: insights into requirements for RNase H cleavage — SUPPLEMENTARY DATA 

# Structures of HIV-1 RT-RNA/DNA ternary complexes with dATP and nevirapine reveal conformational flexibility of RNA/DNA: insights into requirements for RNase H cleavage

## SUPPLEMENTARY DATA

**Files in this Data Supplement:**

- SUPPLEMENTARY DATA
