# Supplementary material for: Structures of HIV-1 RT-RNA/DNA ternary complexes with dATP and nevirapine reveal conformational flexibility of RNA/DNA: insights into requirements for RNase H cleavage
Source: Nucleic Acids Res. 2014 May 31;42(12):8125–37. doi: 10.1093/nar/gku487 (PMC4081091; doi:10.1093/nar/gku487)
Supplement: SUPPLEMENTARY DATA [file supp_gku487_nar-00637-m-2014-File007.pdf]

SUPPLEMENTARY INFORMATION

Structures of HIV-1 RT-RNA/DNA ternary complexes with dATP and nevirapine reveal conformational flexibility of RNA/DNA: insights into requirements for RNase H cleavage

Kalyan Das, Sergio E Martinez, Rajiv P Bandwar, and Eddy Arnold

Center for Advanced Biotechnology and Medicine, Department of Chemistry and Chemical Biology, Rutgers University, Piscataway, New Jersey 08854, USA.

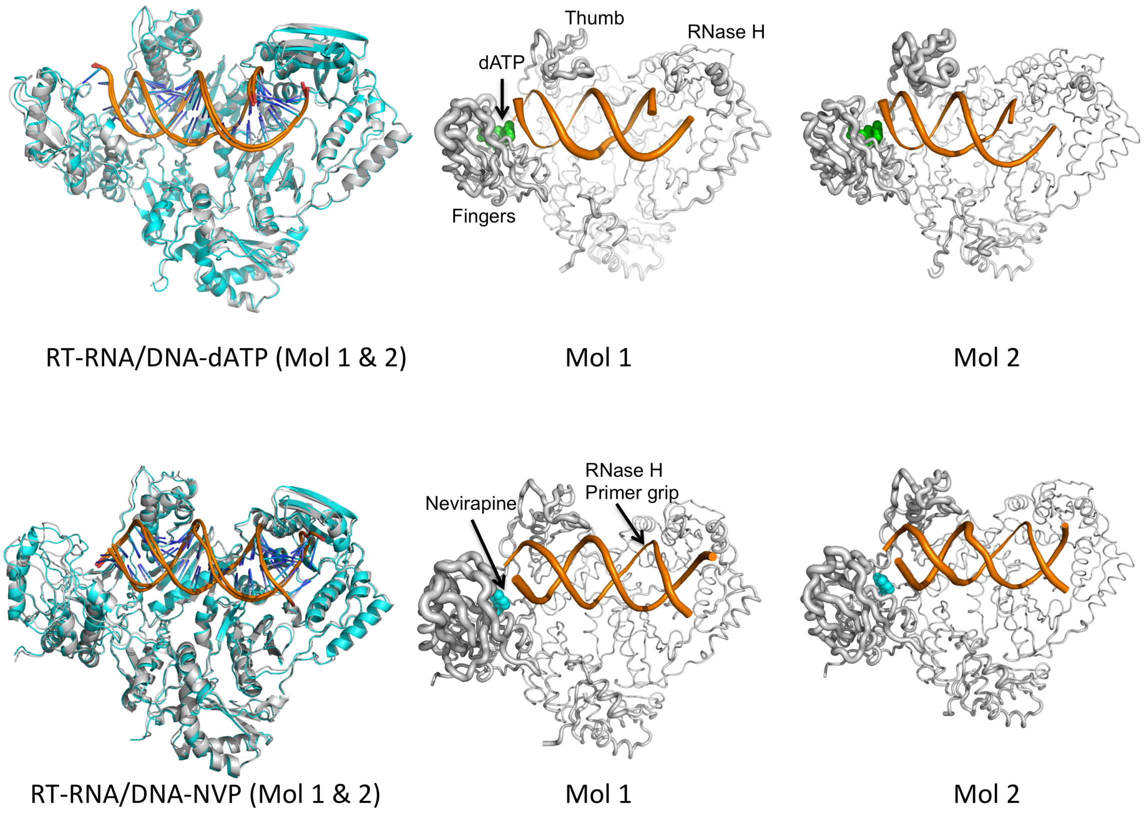

SI Figure 1. Comparison of two copies (Mol-1 and Mol-2) of RT-RNA/DNA-dATP (top) and RT-RNA/DNA-NVP (bottom) complexes in the respective crystallographic asymmetric units. The left figure shows an overall superposition, and the two right figures are plotted based on B-factors of individual residues/nucleotides. Tube thickness is a function of B-factor magnitude: thicker tube represents regions with higher B-factors, i.e., higher flexibility.

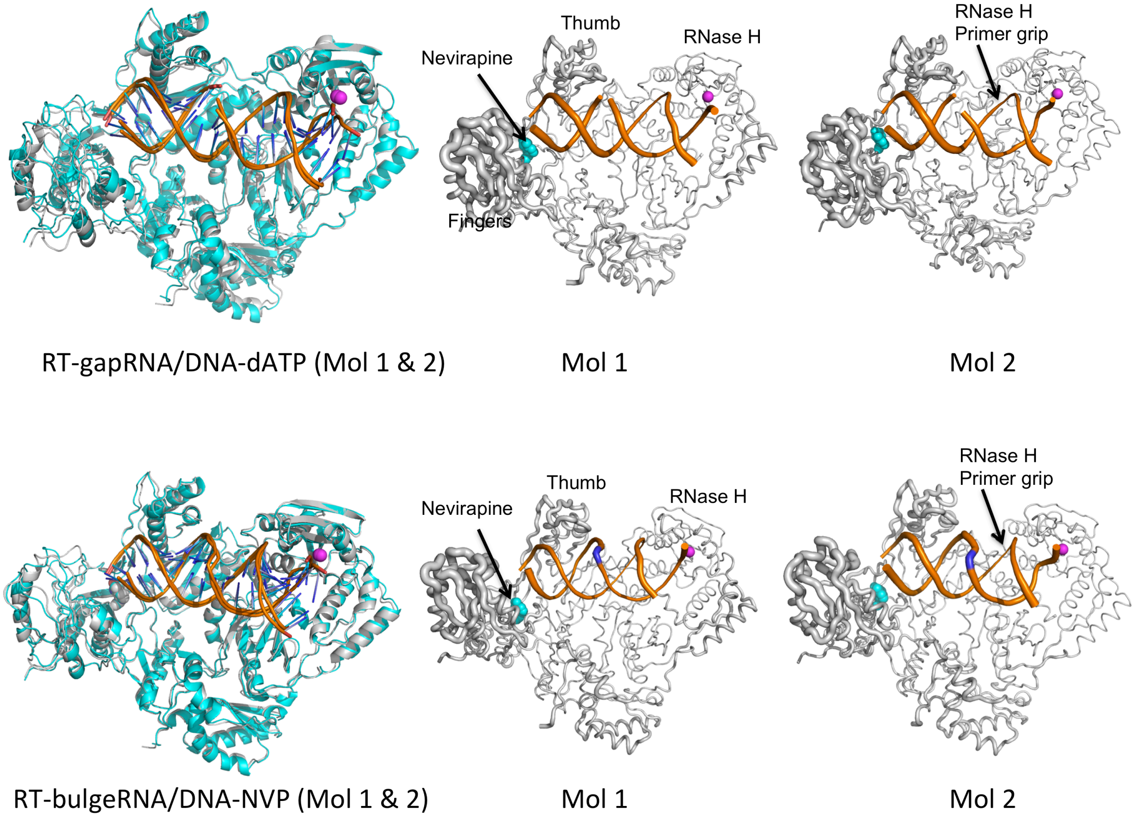

SI Figure 2. Comparison of two copies (Mol-1 and Mol-2) of RT-gapRNA/DNA-NVP (top) and RT-bulgeRNA/DNA-NVP (bottom) complexes in the respective crystallographic asymmetric units. The left figure shows an overall superposition, and the two right figures are plotted based on B-factors of individual residues/nucleotides. Thicker tube represents regions with higher B-factors, i.e., higher flexibility. Observed metal ions (Mn^{2+} or Ca^{2+}) are shown as magenta spheres.

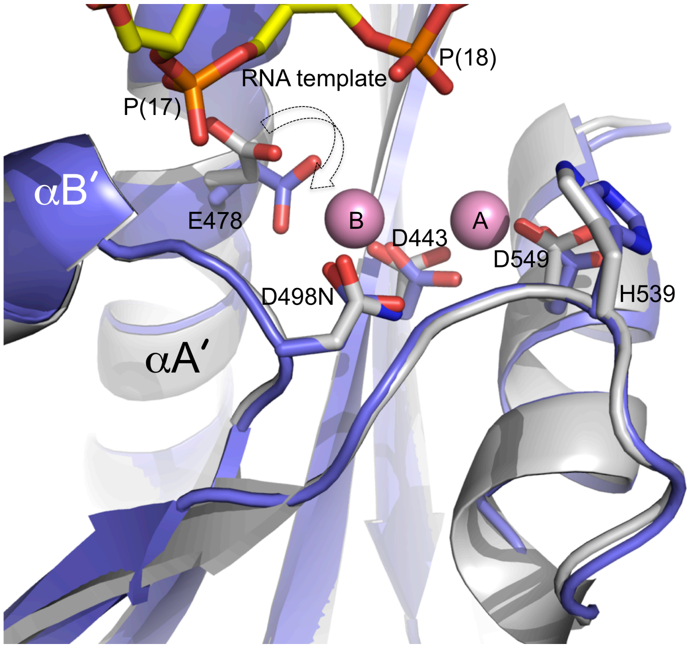

SI Figure 3. Putative structural requirements for chelation of RNA template of an RNA/DNA substrate at the RNase H active site. Superposition of RNase H domains of RT-bulgeRNA/DNA-NVP (protein, gray; template RNA, yellow) and RT-β-thujaplicinol structure (PDB ID 3IG1; blue) (24). The metal ions at the RNase H active site are positioned based on 3IG1 structure. The (i) D498N mutation and (ii) different rotameric conformation of E478 might restrict the binding of metal ion B and chelation of the RNA template in the RT-bulgeRNA/DNA-NVP structure.

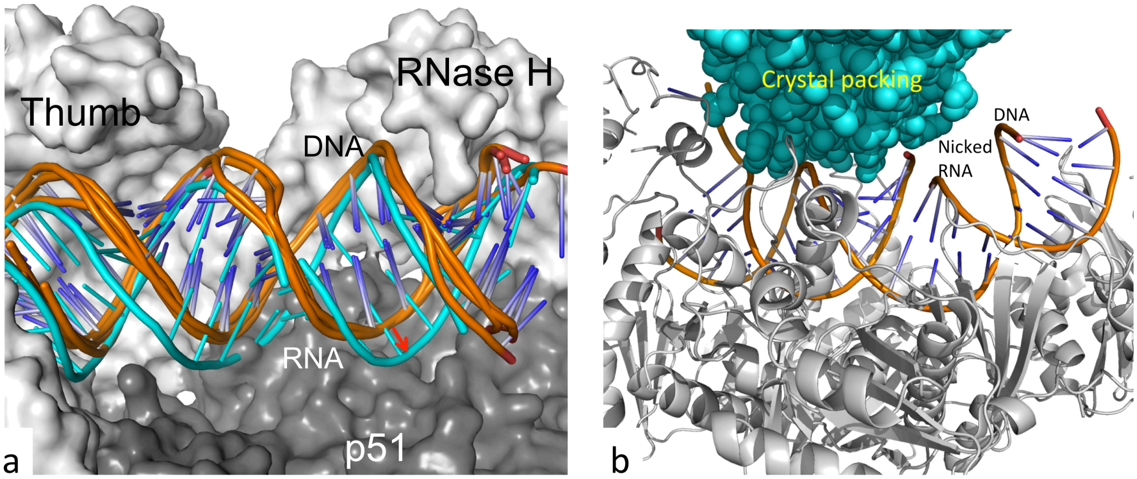

SI Figure 4. A nicked RNA/DNA in RT-RNA/DNA-efavirenz complex (PDB ID 4B3O) (33), that has a different track approaching the RNase H domain, has extensive contacts with an adjacent RT molecule in the crystal. (a) Superposition of RT-nicked RNA/DNA-efavirenz (PDB ID 4B3O; cyan RNA/DNA) onto an array of structures of RT-RNA/DNA-NVP complexes, discussed in the paper. The structures were aligned by Cα superposition of RNase H domains (residues 440 to 550). The red arrow indicates the displacement of the RNA strand in the 4B3O structure approaching the RNase H domain compared to other RT-RNA/DNA-NNRTI structures. (**b**) A symmetry-related RT molecule (cyan CPK model) has extensive interactions with the nicked_RNA/DNA in the 4B3O structure (33). This crystal packing interaction may contribute significantly to the difference in the RNA track, as shown in panel **a**.

SI Table 1. Average inter-base-pair parameters for RT-bound DNA/DNA (D/D) and RNA/DNA (R/D) in crystal structures calculated by CURVES+ (53). The structures of RT-DNA/DNA-AZTTP (or NVP) and RT-RNA/DNA-dATP (or NVP) are used in the calculations, and the parameters for DNA/DNA structures are shaded.

Structure	PDB ID	Shift	Slide	Rise	Tilt	Roll	Twist	H-rise	H-twist
RT-D/D-AZTTP (Mol 1)	3V4I	-0.08	-0.28	3.36	-0.1	6.3	34.0	3.16	34.9
RT-D/D-AZTTP (Mol 2)	3V4I	-0.06	-0.24	3.33	-0.3	6.4	33.6	3.13	34.6
RT-R/D-dATP (Mol 1)	4PQU	0.03	-1.46	3.27	0.7	5.8	31.4	2.96	32.2
RT-R/D-dATP (Mol 2)	4PQU	0.01	-1.49	3.26	0.7	6.4	31.0	2.91	32.0
RT-D/D-NVP (Mol 1)	3V81	-0.16	-0.55	3.32	0.0	5.2	31.6	3.14	32.3
RT-D/D-NVP (Mol 2)	3V81	-0.15	-0.57	3.30	0.1	5.4	32.1	3.12	32.8
RT-R/D-NVP (Mol 1)	4PUO	0.21	-1.38	3.43	3.1	4.1	32.9	3.21	33.9
RT-R/D-NVP (Mol 2)	4PUO	0.25	-1.11	3.47	3.1	6.9	33.7	3.11	35.1
